# Invasive Klebsiella pneumoniae Syndrome in Qatar: A Case Report

**DOI:** 10.7759/cureus.15015

**Published:** 2021-05-13

**Authors:** Muhammad B Jamshaid, Aamir Shahzad, Abdulaziz Zafar, Ijaz Kamal

**Affiliations:** 1 Internal Medicine, Hamad General Hospital, Doha, QAT; 2 Medicine, Hamad General Hospital, Doha, QAT; 3 Internal Medicine, Hamad Medical Corporation, Doha, QAT

**Keywords:** diabetic ketoacidosis (dka), klebsiella pneumonea, disseminated bacteremia, liver abscess aspiration, brain abscess

## Abstract

*Klebsiella pneumoniae (K. pneumoniae)* is a Gram-negative bacteria that can infect most of the body’s organs, from the lungs to the central nervous system. It is notorious for causing pneumonia in alcoholic, diabetic, and hospitalized patients. It is now emerging as a cause of abscesses involving multiple organs. Invasive *K. pneumoniae* is most commonly observed in the Asian population but has been reported in other geographical areas as well. We present a case of invasive *K. pneumoniae*. The patient was initially admitted with diabetic ketoacidosis (DKA); further investigations showed multiple abscesses involving the liver, lungs, brain, and muscles. *K. pneumoniae* was identified in blood and liver abscess cultures. The patient was managed for DKA as per protocol, and was administered broad-spectrum antibiotics with percutaneous drainage of liver abscess for invasive *K. pneumoniae* syndrome. In this paper, we highlight the invasive nature of* K. pneumoniae*, which may aid clinicians in diagnosing and managing similar cases, thereby preventing the associated high morbidity and mortality.

## Introduction

*Klebsiella pneumoniae (K. pneumoniae)* is a Gram-negative encapsulated bacillus that is widely present in nature, including the human oral cavity and intestine [1]. *K. pneumoniae* is the most important organism in the *Klebsiella* genus; it is responsible for hospital-acquired infections in compromised hosts with impaired immune systems. Infections caused by *K. pneumoniae* can be acquired in long-term care facilities, such as nursing homes, and less often, in the community. It can cause various infections, including hospital- and community-acquired pneumonia, bloodstream infections, lung abscesses, empyema, bacteremia, catheter-related infections, wound or surgical site infections, upper and lower urinary tract infections, liver abscesses, and meningitis [2].

*K. pneumoniae* infections occur particularly in patients with chronic alcoholism, diabetes, chronic kidney disease, and those on long-term steroids. Less common infections include endophthalmitis, psoas muscle abscesses, septic arthritis, pyomyositis, and purulent pericarditis [3]. Multiorgan infections occur from an embolic spread of invasive liver abscesses. Embolic *Klebsiella* infection appears to be a rare complication and accounts for only 2% of the total cases [4]. However, it may lead to high morbidity and mortality [5].

## Case presentation

A 40-year-old Nepalese male was brought to the emergency department (ED) by emergency medical services (EMS) due to an episode of acute confusion, headache, and dizziness. He had been recently diagnosed with poorly controlled type 2 diabetes mellitus (HbA1c: 13.6). On examination, the patient was vitally stable; he was aggressive, disoriented to time and place, and had a low level of consciousness, which led to the patient’s intubation in the ED. The emergency head CT was unremarkable. On review of his blood investigations, he was found to have acidosis with a PH of 6.9 (normal range: 7.34-7.45), bicarbonate of 6 mmol/L (normal range: 24-28 mmol/L), and elevated serum beta-hydroxybutyrate level of 6.6 mmol/L (normal value: <0.4 mmol/L). Based on these results, treatment was started for diabetic ketoacidosis (DKA) with IV fluids and IV insulin as per local protocol, and the patient was admitted to the medical intensive care unit (MICU) for further management.

**Figure 1:**
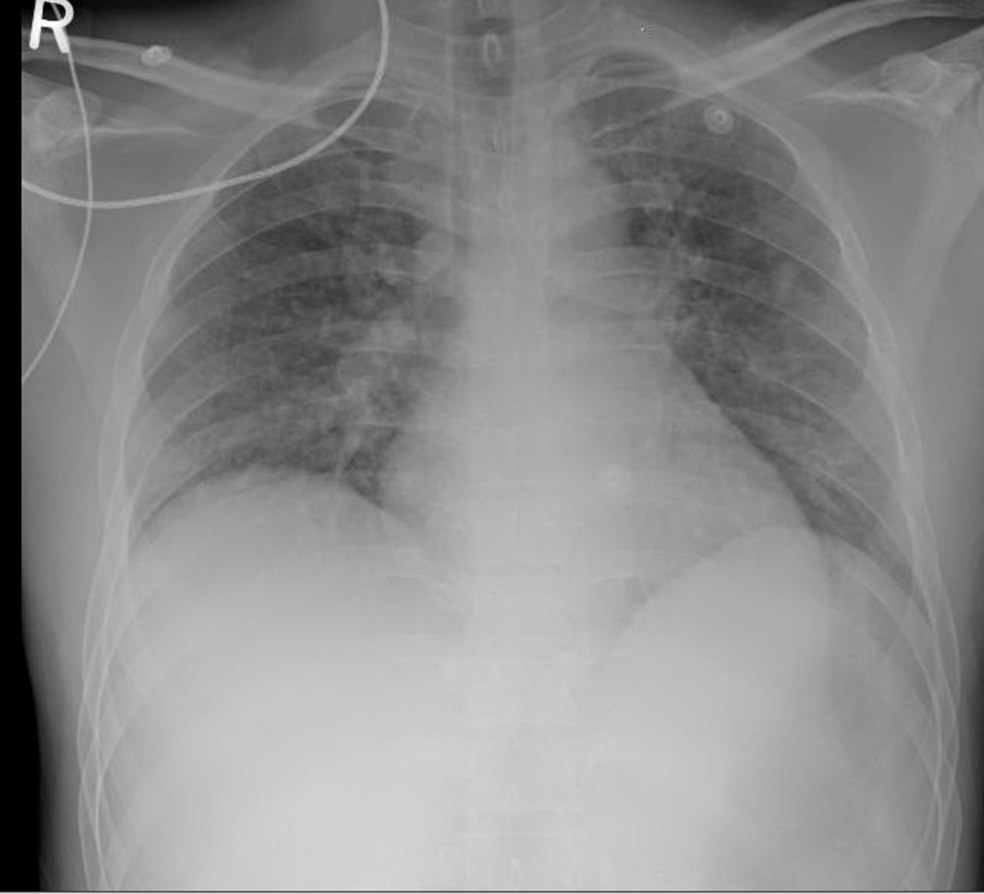
Chest X-ray showing bilateral fluffy infiltrates

Further investigations showed elevated inflammatory markers with leukocytosis. His WBC was 15 x 10^3^/uL (normal range: 4-10 x 10^3^) and C-reactive protein (CRP) was 320 mg/L (normal level: <6 mg/L). After taking samples of blood and urine for culture, the administration of empiric antibiotics piperacillin-tazobactam and clarithromycin were started. A chest X-ray (Figure 1) showed patchy opacities in both lung fields. Later, *K. pneumoniae* was identified in both the blood culture and the tracheal aspirate culture. Antibiogram showed good sensitivity to multiple antibiotics, and hence antibiotic treatment was de-escalated to amoxicillin-clavulanate, as per the local infectious disease protocol.

**Figure 2:**
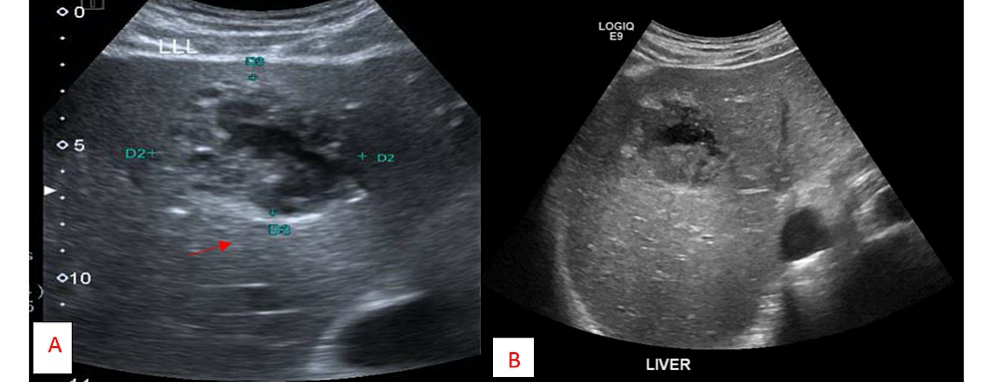
Ultrasound of the abdomen A. Ultrasound of the abdomen showing abscess of dimensions 6.3 x 6 x 5.1 cm and estimated volume of 94 cc. B. Repeat ultrasound performed 15 days after ultrasound-guided abscess drain showing regression in the liver abscess of dimensions 4.4 x 4.8 x 4 cm and estimated volume of 54 cc

On day three, the patient’s condition deteriorated as he got hypotensive, requiring inotropic support. An abdominal ultrasound scan revealed an enlarged liver of 17.7 cm and an ill-defined complex lesion of 6.3 x 6 x 5.1 cm, with radiological findings indicating hepatic abscess (Figure 2A).

**Figure 3:**
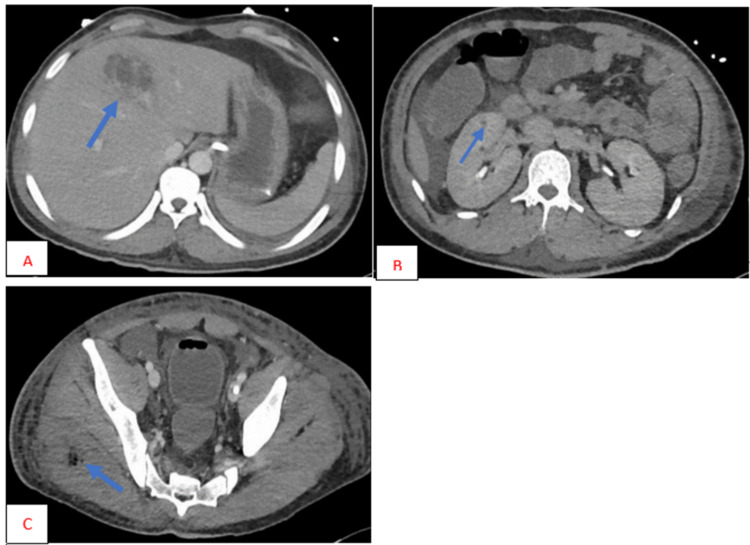
CT of the abdomen The images showing (A) liver abscess, (B) kidney abscess, and (C) gluteal muscle abscess (blue arrows) CT: computed tomography

**Figure 4:**
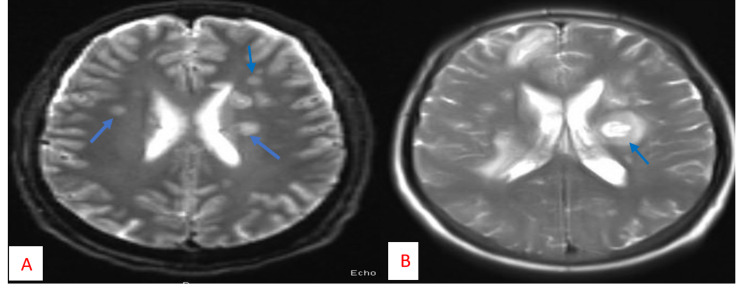
MRI of the head A. First T1Wi MRI head showing bilateral cerebral abscess. B. Repeat T1Wi MRI head 20 days after the first MRI showing new lesions MRI: magnetic resonance imaging

An abdominal CT confirmed the presence of abscesses. It showed an abscess in the liver, multiple small abscesses in both kidneys, and numerous air pockets of abscesses in the right gluteus (Figure 3). During the same day, the patient developed anisocoria. The initial non-contrast head CT was unremarkable. A head MRI showed septic embolic meningoencephalitis with widespread microhemorrhages (Figure 4A). Ultrasound-guided drainage of the liver abscess was performed, and the drained fluid culture was also positive for *K. pneumoniae*. The antibiotics were escalated to meropenem, vancomycin, and metronidazole by the infectious disease team. Transthoracic echocardiogram and transesophageal echocardiogram ruled out any heart valve vegetations. All these tests confirmed that the patient had an embolic spread of *Klebsiella* infection.

On day 10, the patient became hemodynamically stable, which led to the tapering of inotropes and sedation. Despite the administration of multiple antibiotics, the patient continued to have a high-grade fever. An abdominal ultrasound performed 15 days after the ultrasound-guided liver drainage showed interval regression of the liver abscess’s size to 4.4 × 4.8 × 4 cm and an estimated abscess volume of 54 cc (Figure 2B).

After receiving antibiotics for two weeks, the patient’s culture was found to be negative for *K. pneumoniae*. The patient became afebrile and hemodynamically stable, and antibiotics were de-escalated to ceftriaxone and metronidazole. However, the patient’s Glasgow Coma Scale score remained low, and the follow-up head MRI showed a worsening of microabscesses (Figure 3B). Therefore, a tracheostomy was performed, and the patient continued to receive tracheostomy ventilation.

Ceftriaxone and metronidazole were administered for a further six weeks. The patient remained afebrile during this period, and he was shifted to a long-term facility for continuity of care.

## Discussion

Carl Friedlander first identified *K. pneumoniae* in a lung autopsy of a patient who had died from pneumonia in 1882. The bacterium was initially named Friedlander's bacillus. *K. pneumoniae* is considered to be the most common cause of hospital-acquired pneumonia in the United States, and it also accounts for 3-8% of nosocomial infections [6]. The pathogenicity of *K. pneumoniae* depends not only on host factors such as diabetes or chronic alcoholism but also on bacterial virulence [7]. Several virulence factors contribute to the pathogenicity, including hypermucoviscous-specific capsular antigens (i.e., K1 and K2 serotypes) and virulence genes FimH (fimbrial adhesion), rmpA (regulator of mucoid phenotype), uge (uridine-diphosphate galacturonate 4-epimerase), kfu (an iron uptake system), and alls (allantoin metabolism). The K1 and K2 hypermucoviscous-specific capsular serotypes are associated with invasive infections and usually have poor prognoses [7-10].

In our case of embolic *K. pneumoniae*, the risk factor was uncontrolled diabetes mellitus, which is well described in the literature and is very common in the Middle East. Therefore, the Middle East is a particularly high-risk area for *K. pneumoniae* infection, and this issue needs urgent attention. Clinicians must be vigilant to diagnose and manage it effectively and efficiently.

The prognosis for *K. pneumoniae* liver abscess (KLA) is generally good, but embolic complications can cause significant morbidity [7], as in our case, where severe brain damage occurred. KLA with the embolic disease has been predominantly observed in Asia, particularly in Taiwan, but there are case reports from other geographical regions as well [11].

Hypervirulent strains of *K. pneumoniae* can cause a destructive tissue abscess syndrome (e.g., primary liver abscess) with possible metastatic infection. These strains are generally susceptible to cephalosporins, although resistant strains, including those that produce an extended-spectrum β-lactamase (ESBL) or a carbapenem, have been reported before. Appropriate antimicrobial treatment combined with percutaneous drainage of liver abscesses increases the chances of survival for such patients. However, the availability of a facility for early detection of the virulent strain will help in early diagnosis and treatment, which will reduce morbidity and improve outcomes [5].

## Conclusions

We discussed the case of a patient with invasive *K. pneumoniae*. The presentation of *K. pneumoniae* can range from pneumonia to disseminated infection. It should be considered in the differential diagnosis of patients presenting with multiorgan abscesses.
